# Influence of Magnetic Micelles on Assembly and Deposition of Porphyrin J-Aggregates

**DOI:** 10.3390/nano10020187

**Published:** 2020-01-21

**Authors:** Maria Angela Castriciano, Mariachiara Trapani, Andrea Romeo, Nicoletta Depalo, Federica Rizzi, Elisabetta Fanizza, Salvatore Patanè, Luigi Monsù Scolaro

**Affiliations:** 1 CNR-ISMN, Istituto per lo Studio dei Materiali Nanostrutturati c/o Dipartimento di Scienze Chimiche, Biologiche, Farmaceutiche ed Ambientali, University of Messina V.le F. Stagno D’Alcontres, 31 98166 Messina, Italy; mariachiara.trapani@cnr.it (M.T.); anromeo@unime.it (A.R.); 2 Dipartimento di Scienze Chimiche, Biologiche, Farmaceutiche ed Ambientali and C.I.R.C.M.S.B., University of Messina V.le F. Stagno D’Alcontres, 31 98166 Messina, Italy; 3 CNR-IPCF, Istituto Per i Processi Chimico-Fisici, 70124 Bari, Italy; n.depalo@ba.ipcf.cnr.it (N.D.); f.rizzi@ba.ipcf.cnr.it (F.R.); elisabetta.fanizza@uniba.it (E.F.); 4 Dipartimento di Chimica, Università degli Studi di Bari Aldo Moro, Via Orabona 4, 70125 Bari, Italy; 5 Dipartimento di Scienze Matematiche e Informatiche, Scienze Fisiche e Scienze della Terra, University of Messina V.le F. Stagno D’Alcontres, 31 98166 Messina, Italy; salvatore.patane@unime.it

**Keywords:** SPION, porphyrin, chirality, self-assembly, hybrid nanosystems

## Abstract

Clusters of superparamagnetic iron oxide nanoparticles (SPIONs) have been incorporated into the hydrophobic core of polyethylene glycol (PEG)-modified phospholipid micelles. Two different PEG-phospholipids have been selected to guarantee water solubility and provide an external corona, bearing neutral (SPIONs@PEG-micelles) or positively charged amino groups (SPIONs@NH_2_-PEG-micelles). Under acidic conditions and with specific mixing protocols (porphyrin first, PF, or porphyrin last, PL), the water-soluble 5,10,15,20-tetrakis-(4-sulfonatophenyl)-porphyrin (TPPS) forms chiral J-aggregates, and in the presence of the two different types of magnetic micelles, an increase of the aggregation rates has been generally observed. In the case of the neutral SPIONs@PEG-micelles, PL protocol affords a stable nanosystem, whereas PF protocol is effective with the charged SPIONs@NH_2_-PEG-micelles. In both cases, chiral J-aggregates embedded into the magnetic micelles (TPPS@SPIONs@micelles) have been characterized in solution through UV/vis absorption and circular/linear dichroism. An external magnetic field allows depositing films of the TPPS@SPIONs@micelles that retain their chiroptical properties and exhibit a high degree of alignment, which is also confirmed by atomic force microscopy.

## 1. Introduction

Spintronics open a new scenario for the fabrication of efficient devices and refer to electronics based on the electron spin, which is typically controlled by magnetic fields or by ferro-/paramagnetic materials. An important prerequisite for realizing operative spintronic devices is to achieve high spin injection coefficient materials. With this aim, spin filters characterized by the coexistence of one spin conducting and one insulating channel are required. Organic molecules were proposed as a spin filter for “molecular spintronics” due to their relatively longer coherent spin lifetimes and spin transport distances, resulting from weak spin–orbit coupling and weak hyperfine interactions [1,2]. The latter may allow the development of nanoscale devices with improved performance or new functionalities. Porphyrin derivatives, which have already been investigated for molecular electronic devices due to their peculiar electronic and optical properties, have been recently proposed as a class of molecules that is particularly promising as a building block for spintronic technology [3,4]. One-dimensional chromium porphyrin arrays showing half-metallic behavior [5], manganese porphyrin molecules connected with a p-phenylene-ethynylene group [6], and porphyrin/graphene hybrid materials [7] have been reported so far. Theoretical and experimental investigations allowed demonstrating that metallic substrates are able to induce magnetic ordering and the switching of paramagnetic porphyrins, which is due to a superexchange interaction between Fe atoms in the chromophores and Co or Ni atoms in the substrate [8]. Spin-dependent transport properties in an iron-porphyrin such as a carbon nanotube have been investigated, reporting a magnetoresistance ratio that is strongly dependent on the magnetic configuration of the system [9]. Recently, a new promising effective approach for spintronics has emerged using spin selectivity in electron transport through chiral molecules [10,11,12,13,14]. This effect, defined as chiral-induced spin selectivity (CISS), is due to the special property of chiral symmetry that couples the electron spin and its linear momentum acting as a spin filter depending on the handedness of the molecules [15]. The spin-polarized electron current due to the CISS effect can be used to magnetize ferromagnets, potentially allowing the fabrication of less expensive and high-density devices. A DNA double helix [16,17], as well as chiral molecules with (i.e., oligopeptides or chiral polymers) [12,18,19,20,21] or without (helicenes) [22] stereogenic carbon centers have been reported so far. The driving of electrons through chiral layers has been reported for α helix L-polyalanine and CdSe nanocrystals by local light-induced magnetization [23] and for self-assembled monolayers of polyalanine to magnetize a Ni layer [24]. However, the development of this technology is hindered by the fact that solid thin films and nanostructures need a precise control of homogeneity, morphology, and chirality. Spatially uniform chiral films [25,26], supramolecular structures with a programmed helicity [27,28], artificial assemblies [29], and the patterning of chiral nanostructures [30,31] have been developed up to now. These latter showed significant improvements toward technological applications, even if the uniformity and spatial control of the local handedness of chiral self-assembled systems on a surface is still a fundamental open challenge. In this framework, we already investigated the self-assembly of the achiral water soluble 5,10,15,20-tetrakis-(4-sulfonatophenyl)-porphyrin (TPPS) into chiral aggregates on a substrate by combining a wet lithographic method with the local induction of specific chirality imprinted by a chiral templating agent [32]. It is well known that the diacid form of this porphyrin in solution under the opportune experimental conditions is able to self-organize into highly ordered chiral J-aggregates with or without the assistance of chiral species [33,34,35]. Indeed, in the absence of a chiral bias, TPPS could be assembled into chiral aggregates showing a dichroic signal characterized by a positive bisignate Cotton effect [34,36,37,38,39,40,41] whose shape and magnitude is strictly related to the experimental conditions used for the preparation of the aggregates [34,37,42,43,44,45,46]. The formation of chiral supramolecular assemblies from achiral building blocks is a particularly interesting phenomenon [47,48,49,50,51] for the possible correlation with homochirality observed in nature. In many bioprocesses, the interactions between molecules induce a redistribution of the electronic charge accompanied in a chiral system by spin polarization. It has been experimentally demonstrated that this spin polarization adds an enantioselective term to the forces, thus leading to homochiral interaction energies different from heterochiral ones [12]. Herein, we report on the role of organic capped superparamagnetic iron oxide nanoparticles (SPIONs), after their incorporation in the hydrophobic core of polyethylene glycol (PEG)-modified phospholipid micelles (SPIONs@micelles) on the kinetic and spectroscopic behavior of porphyrin J-aggregates, pointing to the formation of a hybrid system. We anticipate that the SPION-loaded micelles are able to efficiently trigger the growth of TPPS J-aggregates under proper experimental conditions and reagent mixing order protocol. Moreover, this assembling strategy allows conjugating the magnetic properties of the SPIONs@micelles with the chiral optical properties of the J-aggregates, thus obtaining hybrid architectures in solution and on solid state by means of an applied magnetic field. By the application of an external magnetic field, SPIONs show magnetization values comparable and magnetic susceptibility much higher than bulk paramagnetic materials [52]. However, they exhibit high coercivity or residual magnetization value with zero magnetic field [53], anisotropy, and large magnetic saturation values [54]. For these magnetic properties and their good biocompatibility, iron oxide nanoparticles (NPs) have been largely reported for biomedical applications [55,56] and in particular as contrast agents for magnetic resonance imaging (MRI) [52,57], in drug delivery [58,59], magnetic hyperthermia [60], magnetically assisted genetic transfection [61], and/or in combined therapeutic and diagnostic use (theranostics) [62]. To the best of our knowledge, no examples of SPIONs involved in the self-assembly of porphyrins in solution and on the solid state of chiral porphyrin aggregates have been reported so far. Besides, iron oxides thin films with an Fe^3+^/Fe^2+^ ratio of 2 have been reported as a promising candidates for spintronic applications, showing high saturation magnetization and magnetic susceptibility [63]. The merging of the NPs magnetic properties and chiroptical properties of the porphyrin aggregates makes the hybrid SPIONs@micelle J-agg assemblies interesting candidates for a variety of potential applications, ranging from optics to electronics and spintronics.

## 2. Materials and Methods

*Chemicals.* Oleic acid (90%), dodecan-1,2-diol (90%), iron pentacarbonyl (98%), oleyl amine (70%), and 1-octadecene (90%) were purchased from Sigma-Aldrich. 1,2-dipalmitoyl-sn-glycero-3-phosphoethanolamine-N-[methoxy (poly(ethylene glycol))-2000] (16:0 PEG-2-PE, ammonium salt) and amine 1,2-distearoyl-sn-glycero-3-phosphoethanolamine-N-[amino(polyethylene glycol)-2000] (DSPE-PEG-amine, ammonium salt) were from Avanti Polar Lipids. The 5,10,15,20-tetrakis-(4 sulfonatophenyl)porphyrin (TPPS) was purchased from Aldrich Chemicals, and its solutions of known concentration were prepared using the extinction coefficient at the Soret maximum (ε = 5.33 × 10^5^ M^−1^cm^−1^ at λ = 414 nm). All the reagents were used without further purification, and the solutions were prepared in dust-free Milli-Q water.

Preparation of PEG-modified phospholipid micelles loaded with SPION. Organic capped SPIONs were synthesized according to the experimental protocols reported in the literature [64]. For the preparation of the micelles loaded with SPIONs and composed only of PEG-2-PE (SPIONs@PEG-micelles), 150 μL of PEG-2-PE in chloroform (3.5 × 10^−2^ M) were mixed with 240 µL of a SPION stock chloroform dispersion (0.08 M); while for the preparation of amine functionalized superparamagnetic micelles (SPIONs@NH_2_-PEG-micelles), 120 μL of PEG-2-PE (3.5 × 10^−2^ M) and 30 μL of DSPE-PEG-amine (3.5 × 10^−2^ M) were used. After the complete evaporation of organic solvent, the SPION/PEG-modified lipid film was treated with 2 mL of phosphate buffer (PBS, 10 mM, pH 7.4). Three consecutive cycles of heating–cooling, at 80 °C and room temperature respectively, were carried out in order to obtain SPION-loaded micelles. The excess of organic capped SPIONs, not eventually encapsulated in the micelles, was removed by mild centrifugation at 5000× *g* for 1 min; the empty micelles were subsequently removed by ultracentrifugation (200,000× *g*) for 16 h. The SPIONs@micelles that were recovered as pellets were resuspended in water, filtered by using 0.2 μm filters (Anotop, Whatman, Merck, Italy), and lyophilized. Water or PBS was used to reconstitute the dried micelles before their characterization or application [65,66].

Aggregation and deposition procedure. TPPS@SPIONs@micelle J-aggregates were prepared in 0.3 M HCl following two different mixing protocol procedures: (i) porphyrin-first protocol (PF), consisting of the addition of a proper volume of acid stock solution to a diluted solution of porphyrin and SPION-loaded micelles, and (ii) porphyrin-last protocol (PL), in which a known amount of porphyrin stock solution is added to diluted magnetic micelles in acidic solution. The concentration of magnetic micelles is reported in terms of the phospholipid monomer concentration. This has been calculated taking into account a weight percentage of about 74% for the phospholipid into the micelles [66]. For the amine-terminated phospholipid, the PEG-PE/DSPE-PEG-amine mixture is in a 4:1 ratio. Glass slides, carefully cleaned with Piranha acidic solution, were immersed into 3 mL of solution containing TPPS@SPIONs@micelle J-aggregates. We used a neodymium magnet cube (1 cm × 1 cm × 1 cm) placed below the glass slides to deposit aggregates onto glass surface from the solution. After an overnight aging time at room temperature, the slides were washed by quick immersion in aqueous acidic solution and dried under a gentle nitrogen flow.

Spectroscopic and morphological characterization. UV-Vis spectra were collected on a diode-array spectrophotometer Agilent model 8452. Kinetic experiments were carried out in the thermostated compartment of the spectrophotometer, with a temperature accuracy of ± 0.1 K. The analysis of the kinetic profiles has been performed by a non-linear fit of the absorption data according to Equation (1):

*E_xt_* = *E_xt_*_∞_ + (E*_xt_*_0_ − *E_xt_*_∞_) (1 + (*m* − 1){*k*_0_*t* + (*n* + 1)^−1^ (*k_c_t*)*^n^*^+1^)^−1/(*m*−1)^ (1)

with *E_xt_*_0_, *E_xt_*_∞_, *k*_0_, *k*_c_, *m* and *n* as the parameters to be optimized or Equation (2):

*E_xt_* = *E_xt_*_0_ + (*E_xt_*_∞_ − *E_xt_*_0_) (exp(−(*kt*)*^n^*)) (2)

with *E_xt_*_0_, *E_xt_*_∞_, *k* and *n* as the parameters to be optimized (*E_xt_*, *E_xt_*_0_ and *E_xt_* are the extinction at time t, at starting time, and at the end of aggregation, respectively). The circular (CD) and linear (LD) dichroism spectra were recorded on a JASCO J-720 spectropolarimeter equipped with a 450 W xenon lamp. The LD spectra under an applied magnetic field have been recorded by setting a couple of neodymium magnets (1 cm × 1 cm × 1 cm) close to the cuvette walls in a perpendicular direction with respect to the light beam. CD and LD spectra were corrected both for the cell and solvent contributions.

Fluorescence emission and resonance light scattering (RLS) experiments were performed on a Jasco mod. FP-750 spectrofluorimeter. A synchronous scan protocol with a right angle geometry was adopted for collecting RLS spectra [67], which were not corrected for the absorption of the samples. Time-resolved fluorescence emission measurements were performed on a Jobin Yvon-Spex Fluoromax 4 spectrofluorimeter using the time-correlated single-photon counting technique. A NanoLED (λ = 390 nm) has been used as the excitation source.

For the transmission electron microscopy (TEM) investigation, a Jeol JEM-1011 microscope, working at an accelerating voltage of 100 kV, was used. TEM micrographs were acquired by an Olympus Quemesa Camera (11 Mpx). A total of 400 mesh amorphous carbon-coated Cu grids were dipped in SPION chloroform dispersion to achieve the sample deposition.

Hydrodynamic particle sizes and size distributions were measured by Dynamic Light Scattering (DLS)and carried out at 25 °C by a Zetasizer Nano-ZS (Malvern Instruments) equipped with a 633 nm He−Ne laser using backscattering detection. Each DLS sample was measured several times, and the results were averaged.

Atomic force microscopy (AFM) and magnetic force microscopy (MFM) measurements were performed using a NT-MDT Smena head working in tapping mode and equipped with a CoCr coated tip (mod. MFM01). To collect the MFM data, the instrument was configured to work in the “double pass AC Magnetic Force” mode. In this working mode, the system produces two images; the first consists of the bare morphology collected line by line in a standard non-contact scanning mode. In the second step, the tip is uplifted at some tenths of a nanometer from the surface, and the phase shift due to the magnetic interaction between the tip and the sample is recorded. During the second step, the profile data collected in the first step are used to assure that the distance between the tip and the sample is constant. To improve the sensitivity, before the measurement, the tip was exposed to the field of a neodymium magnet for a few hours.

## 3. Results

**Figure 1:**
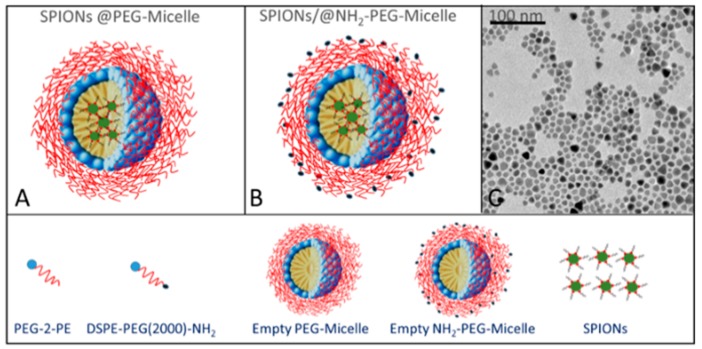
Schematic representation of superparamagnetic iron oxide nanoparticles, after their incorporation in the hydrophobic core of polyethylene glycol (PEG)-modified phospholipid micelles (SPIONs@PEG-micelles) (**A**)- and amine functionalized superparamagnetic micelles (SPIONs@NH_2_-PEG-micelles) (**B**), along with the corresponding legend. TEM micrograph of organic capped SPIONs (**C**).

The TPPS porphyrin is present in neutral aqueous solution as free base characterized by the presence of a Soret band centered at 414 nm and four Q-bands between 500 and 700 nm in the UV/Vis spectrum. Under acid conditions, the protonation of the pyrrole nitrogen atoms of the central core occurs (pKa: approximately 4.9), with the formation of a protonated species showing a Soret band at 434 nm and two Q-bands. This diacid specie is able to self-arrange in J-aggregates characterized by a linear arrangement of the chromophores and stabilized by electrostatic interactions between the negatively charged benzenesulfonate groups and the positively charged nitrogen atoms of the pyrrole rings, as well as by hydrogen bonds and stacking interactions [41,68,69,70,71,72,73,74]. The formation in solution of these aggregates leads in the absorption spectrum to the formation of a new band, bathochromically shifted (Δλ: approximately 50 nm) with respect to the monomeric species. Their aggregation kinetics can be influenced by different experimental parameters such as the nature of the acid [45], the ionic strength [41,74], or the reagent mixing order protocol [34,72,73]. In particular, this latter influences the dynamics of the growth and eventually both the morphology and size of the assemblies [73]. Here, we investigated on the ability of SPION-loaded micelles to efficiently promote the formation of hybrid TPPS@SPIONs@micelle J-aggregates. In particular, two different magnetic micelles were prepared to investigate the self-assembly process. The aqueous dispersibility of the hydrophobic SPIONs was guaranteed by the formation of SPIONs encapsulating micelles composed of 1,2-dipalmitoyl-sn-glycero-3-phosphoethanolamine-N-[methoxy (poly(ethylene glycol))-2000] (PEG-2-PE), or a mixture of PEG-2-PE and amine 1,2-distearoyl-sn-glycero-3-phosphoethanolamine-N-[amino(polyethylene glycol)-2000] (DSPE-PEG-amine], bearing PEG chains and amine terminated PEG chains, respectively (Figure 1) [66,75]. Indeed, magnetic micelles composed only of PEG-2-PE (SPIONs@PEG-micelles, Figure 1A) and amine-functionalized magnetic micelles based on a PEG-2-PE/DSPE-PEG-amine mixture (SPIONs@NH_2_-PEG-micelles, Figure 1B) were obtained by the encapsulation of a certain number of organic-capped SPIONs having an average diameter of about 9 nm (Figure 1C) clustered in the hydrophobic core into the single micelle.

**Figure 2:**
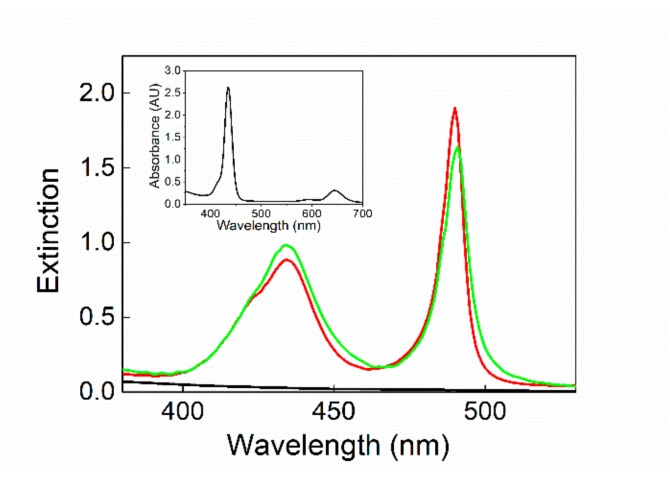
UV-vis spectra of SPIONs@PEG-micelles (black line) and chiral J-aggregates formed by 5,10,15,20-tetrakis-(4-sulfonatophenyl)-porphyrin (TPPS) embedded into the magnetic micelles (TPPS@SPIONs@PEG-micelles) in aqueous solution, porphyrin first (PF) (red line) and porphyrin last (PL) mixing protocol (green line). Experimental conditions: [SPIONs@PEG-micelles] = 15 μM, [TPPS] = 5 μM, [HCl] = 0.3 M. For comparison, TPPS@SPIONs@PEG-micelles at pH 3 is reported in the inset.

DLS measurement was carried out on SPIONs@PEG-micelles to test their stability. From the analysis of the data, the micelles both in neutral and acidic aqueous solution show an average R_H_ of 150 (±10) nm. When SPIONs@PEG@micelles (15 μM) were added to a solution of TPPS at neutral or mild acidic pH, no evidence of modification in the spectroscopic behavior of the chromophore, in terms of absorption (inset of Figure 2), fluorescence emission, fluorescence lifetimes, and time-resolved fluorescence anisotropy has been observed (data not shown). The experimental evidence excludes a preinteraction among porphyrins and magnetic micelles before aggregation. In order to promote the formation of supramolecular assemblies among porphyrin aggregates and magnetic micelles, we lowered the pH (0.3 M HCl) following the two previously mentioned mixing order protocols. Using a PF protocol, although the J-aggregates electronic spectrum remains unchanged (Figure 2), their formation kinetics are conversely deeply influenced by the presence of SPIONs@PEG-micelles. A kinetic analysis of the extinction/time traces on the aggregate band (491 nm) has been performed by using an autocatalytic model already reported in the literature and largely reported for kinetic investigations on porphyrin aggregation [34,39,44,45,76].

**Figure 3:**
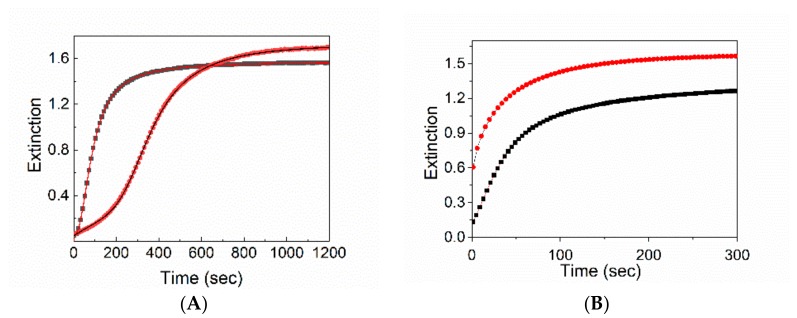
Extinction kinetic traces at 491 nm with (black line) and without (red line) SPIONs@PEG-micelles. The solid lines are the best-fitting of the experimental data (λ_491 nm_) to Equation (1) (**A**) and Equation (2) (**B**). The best-fitting parameters are collected in Table 1. Experimental conditions: [SPIONs@PEG-micelles] = 15 μM, [TPPS] = 5 μM, [HCl] = 0.3 M PF, *T* = 298 K mixing order protocol (**A**), PL mixing order protocol (**B**).

The kinetic profiles exhibit a sigmoidal behavior characterized by the presence of an initial induction period that was shortened in the presence of magnetic micelles, (Figure 3A) with an observed rate constant *k*_c_ that is about an order of magnitude larger than that observed for the pure system. All the kinetic parameters are summarized in Table 1.

**Figure 4:**
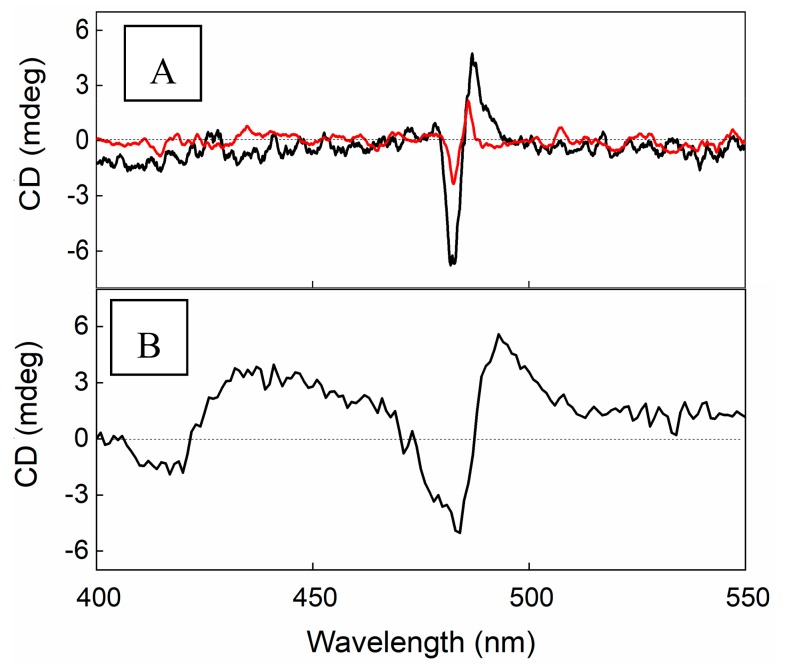
Circular dichorism (CD) spectra of J-aggregates in solution (**A**) and on solid state (**B**) in the presence (black line) or in the absence (red line) of SPIONs@PEG-micelles. Experimental conditions: [SPIONs@PEG-micelles] = 15 μM, [TPPS] = 5 μM, [HCl] = 0.3 M, PL mixing order protocol.

An increase of the aggregation kinetic rate has been observed also for the PL mixing order protocol. According to the literature, the PL protocol induces a dramatic difference in the kinetic profiles, which now obey a stretched exponential form (Equation (2)). The presence of magnetic micelles further increases the rate constants of the aggregation process (k), whose value goes from 1.8 × 10^−2^ s^−1^ to a 2.7 × 10^−2^ s^−1^ (Figure 3B). It is noteworthy that unlike the previous experimental protocol, in this case, the electronic spectrum of the final aggregates shows a slight bathochromic shifted and widened J-absorption (Figure 2). DLS experiments point to the presence in solution of particles having a *R_H_* value of approximately 1 µm. Since it is known that TPPS aggregates in solution in analogous experimental conditions are nanorods sizing hundreds of nanometers [41], our experimental findings suggest the formation of a hybrid system formed by porphyrin aggregates and magnetic micelles. As TPPS J-aggregates can undergo spontaneously to symmetry breaking, circular dichroism spectra have also been collected. At the end of aggregation kinetics for the hybrid system obtained by PL protocol, the CD spectrum shows a profile with a positive bisignate Cotton effect centered at the aggregate absorption band that is much higher with respect to the neat sample (Figure 4).

**Figure 5:**
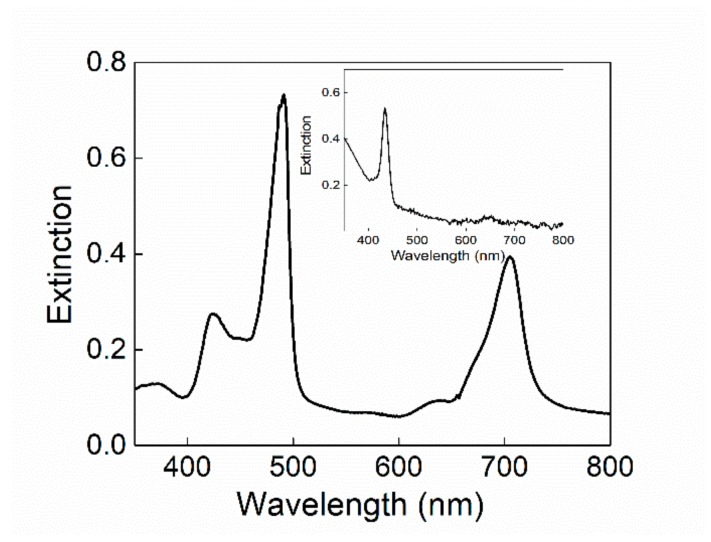
Extinction spectrum of TPPS@SPIONs@PEG-Micelle J-aggregates deposited on the glass surface by applying a magnetic field through a neodymium magnet cube 1 cm × 1 cm × 1 cm and residual specie in solution (inset). Experimental conditions: [SPIONs@PEG-Micelles] = 15 μM, [TPPS] = 5 μM, [HCl] = 0.3 M, PL mixing order protocol.

It is interesting to note that according to the literature, for “pure” TPPS J-aggregates, whatever the pretreatment of the samples, the dissymmetry g-factor generally decreases on increasing the value of the kinetic rate constant [44]. On the contrary, for the hybrid system here reported, we observe an increase on the CD intensity signal, despite the increase of the aggregation rate constant. Taking advantage of the magnetic properties of the SPIONs@PEG-micelles, we used a magnet field below the sample to try to deposit aggregates obtained by PF and PL protocols, respectively, onto glass surface from the solutions. After aging overnight at room temperature, the formation of a green film from the PL samples and of a dark orange precipitate from the PF protocol samples respectively, is evident. As a blank experiment, the magnet field has been applied also for the aggregate solutions in the absence of magnetic micelles and, as expected, no deposition has been observed. The extinction spectrum of the solid sample shows unequivocally the presence of the characteristic J-aggregate band (Figure 5), whereas in solution, only the presence of porphyrin in its diacid monomeric form has been observed (Figure 5, inset). Interestingly, the CD spectrum of the film shows the chiroptical properties observed in solution with an induced bisignate Cotton effect in the aggregate absorption region (Figure 4B). This dichroic signal cannot be ascribed to linear dichroism, as no variation due to the sample orientation with respect to incident light has been observed.

These experimental findings prove the formation of a hybrid system only for the PL protocol. So, in our case, the reagent mixing order affects not only the kinetic rates of the self-assembly processes but also the formation of the hybrid supramolecular system. This sort of “YES/NO” effect has already been reported for TPPS J-aggregates obtained in the presence of polyamines containing less than three protonable nitrogen atoms [73]. In particular, the nucleation step is the critical parameter affecting both the kinetic and mesoscopic structure of the resulting aggregates [34,77]. In the present case, we hypothesize that the PL protocol, due to a concentration effect, induces a very quick formation of a larger number of porphyrin seeds that can be entrapped in peripheral hydrophilic chains of the SPIONs@PEG-micelles, leading to hybrid aggregates. On the contrary, in the PF protocol, a longer nucleation period allows the organization of porphyrins in much larger aggregates, thus preventing their entrapment in the magnetic micelles.

**Figure 6:**
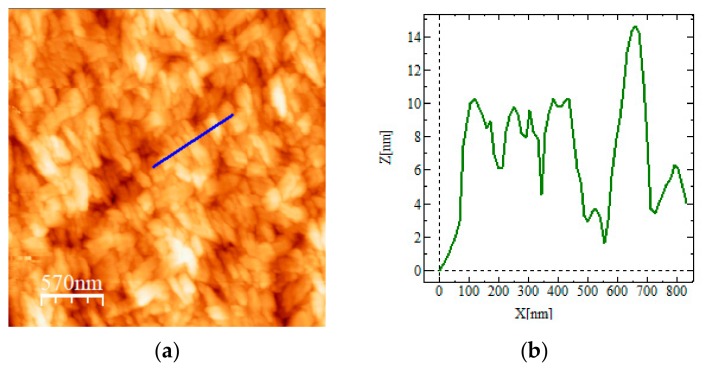
Atomic force microscopy (AFM) topography images (**a**) and relative profile (**b**) of the TPPS@SPIONs@PEG-micelle J-aggregates deposit on a glass substrate after applying a magnetic field.

In order to characterize the morphology of the deposited sample on a glass surface, we performed atomic force microscopy (AFM). Figure 6 shows the sample topography and the line profile acquired along the blue line, as depicted on the picture. The morphology consists of an almost homogeneous layer of compactly arranged nanostructures, whose dimension ranges between 100 and 150 nm. It is worth noting that a preferential direction is present; this alignment is probably due to the magnetic field used during the deposition method. Even if no clear detection of the TPPS aggregates is possible, their embedding into the film is confirmed by the absorption of the sample (Figure 5).

**Figure 7:**
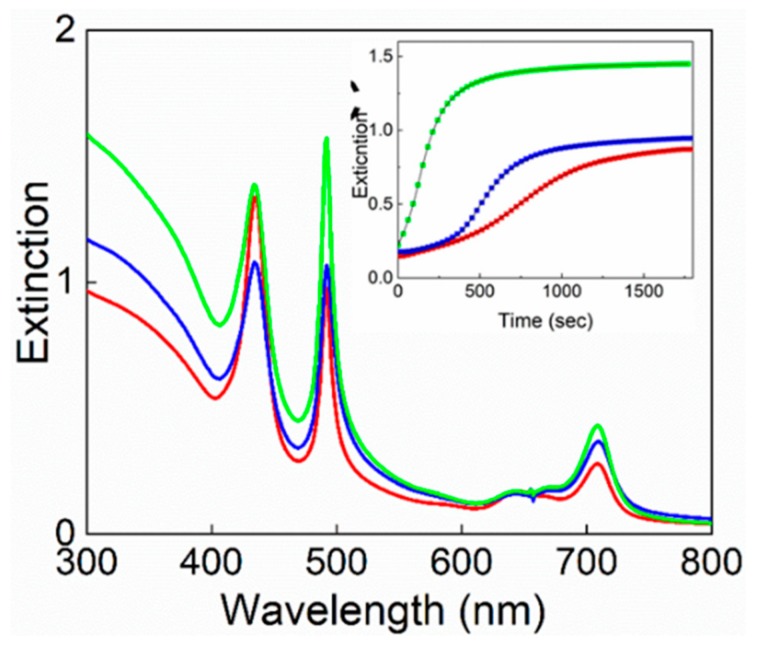
Extinction spectra for TPPS@SPIONs@NH_2_-PEG-micelles J-aggregates formed at different SPIONs@NH_2_-PEG-micelles concentrations. [SPIONs@NH_2_-PEG-micelles] = 15 μM (red line), [SPIONs@NH_2_-PEG-micelles] = 22 μM (blue line), [SPIONs@NH_2_-PEG-micelles] = 45 μM (green line). In the inset, the extinction kinetic traces at 491 nm and the best fitting experimental data according to Equation (1). Experimental conditions: [TPPS] = 5 μM, [HCl] = 0.3 M PF mixing order protocol, *T* = 298 K.

As the interaction among TPPS porphyrins and functional molecules bearing protonable nitrogen atoms such as polyamines or PAMAM dendrimers has been already reported in aqueous solution [72,73,78,79,80], we exploited amine functionalized magnetic micelles based on a PEG-2-PE/DSPE-PEG-amine mixture (SPIONs@NH_2_-PEG-micelles) to trigger the aggregation process. The presence of protonable nitrogen atoms in the periphery of the micelles should be useful for interaction with the negatively charged groups present on the porphyrin ring. Accordingly, we tested the preinteraction between the chromophoric unit in its monomeric neutral and diacid forms with SPIONs@NH_2_-PEG-micelles at different stoichiometric ratios. In all the investigated samples, no changes on the spectroscopic behavior of the porphyrin have been observed so excluding the presence of the chromophoric units embedded in the micelles or entangled in their peripheral chains, even if some interaction with the peripheral counter-cations cannot be ruled out. As for the previous system, the stability of the SPIONs@NH_2_-PEG-micelles under the investigated experimental conditions has been tested by UV/Vis and DLS measurements. This latter technique evidences the presence in solutions of objects with R_H_ of about 140 (±10) nm. The aggregation process in the presence of TPPS has been induced by employing the two different mixing order protocols, PF and PL, respectively. When PL protocol is adopted on SPIONs@NH_2_-PEG-micelles under acidic conditions (HCl 0.3 M), J-aggregates form almost instantaneously. The resulting solutions are highly unstable, and precipitation occurs in a very short time. DLS investigations confirm the presence of micrometric objects, thus justifying the experimental observations. Due to the scarce reproducibility of these samples, we decided to not further investigate them. On the contrary, when PF protocol is used, the aggregation is fostered by the addition of acidic solution of SPIONs@NH_2_-PEG-micelles at different concentrations; the extinction and kinetic features are reported in Figure 7. In particular, the inset shows a set of kinetic traces obtained from the extinction increase at 491 nm (Figure 7). These curves display a sigmoidal profile characterized by a nucleation early stage, which shortens upon increasing the concentration of magnetic micelles in solution (inset Figure 7). The values of the parameter *m* (~3), that is the critical size for the nuclei, and n (~3−4), the time exponent, are comparable to those reported in the literature for similar systems in aqueous solutions [34]. Differently, the values of the rate constants for the uncatalyzed pathway, *k*_0_, and those for the catalyzed pathway, *k_c_*, in the presence of magnetic micelles are higher with respect to the pure self-assembled system and increase, exhibiting an exponential dependence on SPIONs@NH_2_-PEG-micelles concentration. All the kinetic parameters are collected in Table 1. After equilibration, the UV–Vis spectra provide evidence that the amount of the final aggregate increases with the concentration of SPIONs@NH_2_-PEG-micelles.

**Figure 8:**
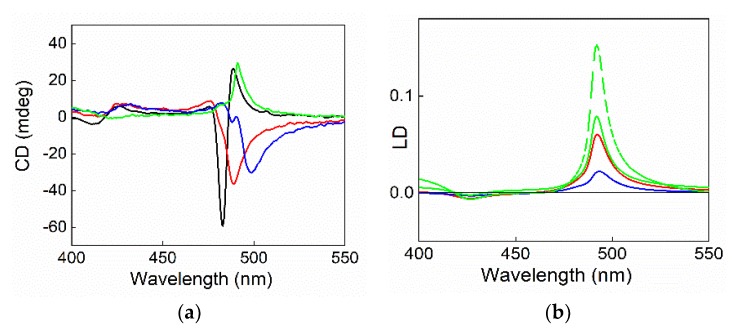
CD (**a**) and linear dichorism (LD) (**b**) spectra for TPPS@SPIONs@NH_2_-PEG-micelles J-aggregates at different SPIONs@NH_2_-PEG-Micelles concentrations. In the absence of SPION-NH_2_ (black line) [SPIONs@NH_2_-PEG-Micelles] = 15 μM (red line), [SPIONs@NH_2_-PEG-Micelles] = 22 μM (blue line), [SPIONs@NH_2_-PEG-Micelles] = 45 μM (green line), and under an applied magnetic field perpendicularly to the light path (green dashed line). Experimental conditions: [TPPS] = 5 μM, [HCl] = 0.3 M, PF mixing order protocol.

The aggregated samples are stable in solution, and DLS measurements show the presence of objects with R_H_ values of about 700 (±50) nm. The CD spectra recorded at the end of the aggregation process show in the absence of magnetic nanoparticles the typical positive bisignate spectrum, whereas unusual profiles can be observed in the presence of SPIONs@NH_2_-PEG-micelles at different porphyrin/magnetic micelles concentration ratios (Figure 8a). A generally inverted and consistently bathochromically shifted band is present at the absorption of the J-component, while the H-band shows the usual positive bisignate feature, even if it is slightly red shifted. Since linear dichroism (LD) could be responsible for the observed effects in the case of alignment of the aggregates, we performed LD measurements on the same TPPS@SPIONs@NH_2_-PEG-micelles J-aggregates (Figure 8b). The LD spectra show for all the samples the presence of a positive band at 491 nm and a less intense and negative one at 420 nm, similarly to those reported for analogous systems, where the J-aggregates were aligned by flow. The presence of different signs for the two LD bands has been attributed to the different polarization within the porphyrin aggregate of the J and the H electronic transitions [81].

**Figure 9:**
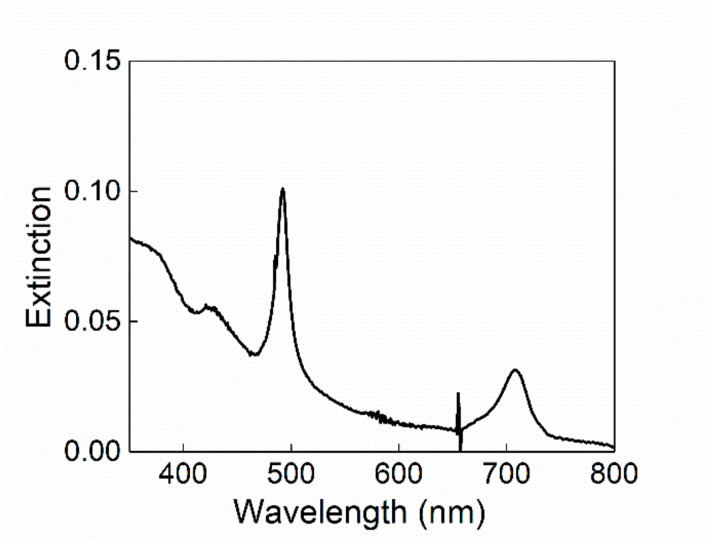
Extinction spectrum of TPPS@SPIONs@NH_2_-PEG-micelles J-aggregates deposited on a glass surface by applying a magnetic field under the solid substrate. Experimental conditions: [SPIONs@NH_2_-PEG-micelles] = 45 μM, [TPPS] = 5 μM, [HCl] = 0.3 M, PF mixing order protocol.

This experimental evidence suggests that the obtained aggregates being large enough are somehow aligned, probably by effect of barodiffusion. Taking advantage of the magnetic property of TPPS@SPIONs@NH_2_-PEG-micelles J-aggregates, a further LD spectrum was taken on the solution containing the larger amount of NPs by applying an external magnetic field perpendicularly to the direction of the optical beam during the measurement. In comparison with the LD spectrum recorded at zero field (Figure 8b, full green line), the profile in the presence of the magnetic field exhibits a twofold increase in the intensity of the linear contributions with no alteration of the shape (Figure 8b, dashed green line), thus suggesting an enhanced alignment effect. All our experimental findings point to the formation of a hybrid system, evidencing the role of the magnetic NPs on their kinetics of growth and final structure. In this case, also deposition on a glass surface has been achieved by applying a magnetic field, and the electronic spectrum of the film shows the presence of the J-aggregates band (Figure 9).

**Figure 10:**
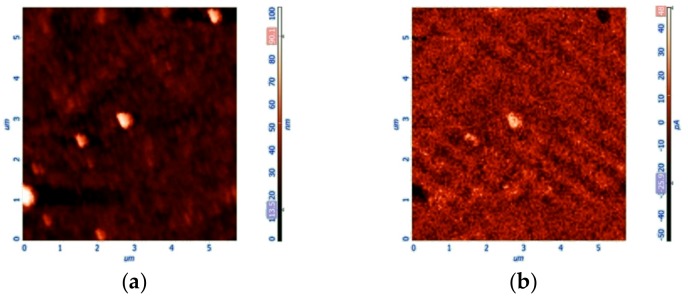
AFM images for TPPS@SPIONs@NH_2_-PEG-micelles J-aggregates deposited on glass by applying a magnetic field under the solid substrate. Topographic (**a**) and magnetic (**b**) images. Experimental conditions: [SPIONs@NH_2_-PEG-Micelles] = 45 μM, [TPPS] = 5 μM, [HCl] = 0.3 M, PF mixing order protocol.

Figure 10 displays the morphology (a) and the magnetic response (b) of this sample obtained through MFM. The morphology consists of an almost homogeneous compact layer of nanoparticles. The surface is also populated by a small number of aggregates with larger sizes. The MFM map reveals a general magnetic activity, which is highlighted by the signal modulation distributed over almost all the sample surface, as the intrinsically limited resolution of the technique is not able to map the magnetic response of the smallest nanoparticles. The bigger aggregates clearly show a much stronger magnetic signal. Interestingly, some particles show a bright contrast, while others highlight a dark contrast. This behavior is due to the different interaction with the magnetic tip that operates in tapping mode at its resonance frequency: a repulsive magnetic force gradient will cause the resonance curve to shift to a higher frequency, an increase in phase shift, and a bright contrast. Conversely, an attractive magnetic force gradient results in the resonance curve shifting to a lower frequency, and a decrease in phase shift resulting in a dark contrast. Some particles with no magnetic activity are also observed. Therefore, all the spectroscopic and microscopy evidence suggests that the deposited film is constituted by the hybrid TPPS@SPIONs@NH_2_-PEG-micelles J-aggregates.

## 4. Conclusions

J-aggregates of TPPS porphyrin are interesting nanomaterials, since depending on the experimental conditions and templating agents, they can be prepared in a variety of different morphologies. The use of magnetic micelles offers a way to obtain hybrid organic/inorganic nanosystems that conjugate the properties of each constituent component. The porphyrin nanoaggregates exhibit peculiar extinction bands, with enhanced resonant light-scattering effects [34], and chiroptical properties that can spontaneously appear or be induced by doping them with a proper chiral reagent [40]. The SPIONs micelles, with the two different capping phospholipids, while allowing for the water solubility of the inorganic cluster core, afford a hydrophilic surface where eventually TPPS can nucleate, leading to the final J-aggregates. The presence of an amino-terminated chain in the case of SPIONs@NH_2_-PEG-micelles determines a distinct change in the supramolecular assembling kinetics of TPPS. Furthermore, the nature of the terminal PEG chain on the phospholipid influences the way in which the two different mixing protocols control the formation of stable nanosystems. In both cases, taking advantage of the magnetic properties of SPIONs, it is possible to drive the deposition of TPPS J-aggregates embedded into the micelles onto surface, thus achieving a high level of alignment into the film. Considering the optical and chiroptical response, still conserved in the solid phase, and their magnetic properties, we expect that these nanosystems could find potential applications in a variety of fields, including spintronics, optoelectronics and theranostics.

## Figures and Tables

**Table 1:** Kinetic parameters *k*_0_, *k_c_*, *m,* and *n* for the aggregation of TPPS@SPIONs@PEG-micelles and TPPS@SPIONs@NH_2_-PEG-micelles as a function of the mixing order protocol and/or magnetic micelles concentration (*T* = 298 K). [a] Data obtained according to Equation (2); [b] Data obtained according to Equation (1).

J-Aggregates	*K*		*N*	
[SPIONs@PEG-micelle]/µM [a]	−		−	
−	1.8 × 10^−2^ ± 1 × 10^−4^		0.8 ± 0.05	
15	2.7 × 10^−2^ ± 1 × 10^−3^		0.7 ± 0.02	
	***k*_0_**	***k_c_***	***m***	***n***
[SPIONs@PEG-micelle]/µM [b]	−	−	−	−
0	2.7 × 10^−4^ ± 7 × 10^−6^	1.9 × 10^−3^ ± 8 × 10^−6^	3.0 ± 0.1	4.0 ± 0.1
15	−	1.6 × 10^−2^ ± 4 × 10^−5^	2.3 ± 0.1	1.3 ± 0.1
[SPIONs@NH_2_-PEG-micelle]/µM[b]	−	−	−	−
0	2.7 × 10^−4^ ± 7 × 10^−6^	1.9 × 10^−3^ ± 8 × 10^−6^	3.0 ± 0.1	4.0 ± 0.1
15	4.1 × 10^−4^ ± 2 × 10^−7^	1.7 × 10^−3^ ± 9 × 10^−6^	2.2 ± 0.1	3.6 ± 0.3
22	1.2 × 10^−4^ ± 5 × 10^−6^	2.6 × 10^−3^ ± 5 × 10^−6^	3.0 ± 0.3	4.3 ± 0.1
45	2.5 × 10^−3^ ± 7 × 10^−7^	9.1 × 10^−3^ ± 8 × 10^−6^	3.5 ± 0.2	3.0 ± 0.2
